# Combination strategies to optimize the efficacy of chimeric antigen receptor T cell therapy in haematological malignancies

**DOI:** 10.3389/fimmu.2022.954235

**Published:** 2022-08-23

**Authors:** Xinyi Xiao, Yazhuo Wang, Zhengbang Zou, Yufei Yang, Xinyu Wang, Xin Xin, Sanfang Tu, Yuhua Li

**Affiliations:** ^1^ The Second School of Clinical Medicine, Zhujiang Hospital, Southern Medical University, Guangzhou, China; ^2^ School of Rehabilitation Sciences, Southern Medical University, Guangzhou, China; ^3^ Department of Haematology, Zhujiang Hospital, Southern Medical University, Guangzhou, China; ^4^ Bioland Laboratory (Guangzhou Regenerative Medicine and Health Guangdong Laboratory), Guangzhou, China

**Keywords:** CAR T cell, resistance, relapse, combination therapy, chemotherapy, radiotherapy, haematological stem cell transplantation, targeted therapy

## Abstract

Chimeric antigen receptor (CAR) T cell therapy has revolutionized the therapeutic landscape of haematological malignancies. However, resistance and relapse remain prominent limitations, and they are related to the limited persistence and efficacy of CAR T cells, downregulation or loss of tumour antigens, intrinsic resistance of tumours to death signalling, and immune suppressive microenvironment. Rational combined modality treatments are regarded as a promising strategy to further unlock the antitumor potential of CAR T cell therapy, which can be applied before CAR T cell infusion as a conditioning regimen or in *ex vivo* culture settings as well as concomitant with or after CAR T cell infusion. In this review, we summarize the combinatorial strategies, including chemotherapy, radiotherapy, haematopoietic stem cell transplantation, targeted therapies and other immunotherapies, in an effort to further enhance the effectiveness of this impressive therapy and benefit more patients.

## Introduction

Chimeric antigen receptor (CAR) T cell therapy is one of the major breakthroughs in the field of cancer immunotherapy. Remarkably, it has greatly improved the prognosis of haematological malignancies, and six CAR T cell products have been approved by the U.S. Food and Drug Administration (FDA) for relapsed/refractory B cell non-Hodgkin lymphoma (R/R B-NHL), R/R B cell acute lymphoblastic leukaemia (B-ALL), and R/R multiple myeloma (MM) **(** **Table 1** **)**. Although CAR T cell products are commercially available, the issues of primary resistance and relapse remain. In B-ALL, 80% of patients can achieve CR after CD19 CAR T cell therapy, whereas approximately 30-50% of patients relapse, the majority within 1 year after infusion (13). The primary efficacy of CAR T cells in B-NHL and MM is comparatively inferior, with 54% and 44.8% CR, respectively (18, 19). There is still significant room to improve the efficacy of CAR T cell therapy.

**Table 1:** Pivotal trials and real-world data of FDA approved CAR T cell products.

Product	Trial	Area	N	ORR	CR	Survival
**LBCL**						
Axi-cel	ZUMA-1^a^ (1)	US	101	82%	54%	PFS 41% (15m); OS 52% (18m)
	Nastoupil et al. (2)	US	275	82%	64%	PFS 47% (1y); OS 68% (1y)
	Pasquini et al. (3)	US	295	70%	52%	NR
	Baird et al. (4)	US	41	88%	66%	Median PFS 6.1m
	Jacobson et al. (5)	US	122	70%	50%	Median PFS 4.5m; OS 67% (1y)
	Kuhnl et al. (6)	UK	224	77%	52%	PFS 42% (1y); OS 57% (1y)
Tisa-cel	JULIET^a^ (7)	Global	93	52%	40%	Median OS 1y; RFS 65% (1y)
	Pasquini et al. (8)	US & CAN	155	62%	40%	PFS 39% (6m); OS 71% (6m)
	Iacoboni et al. (9)	Spain	75	60%	32%	Median OS 10.7m; PFS 32% (1y)
	Kuhnl et al. (6)	UK	76	57%	44%	PFS 27% (1y); OS 44% (1y)
Liso-cel	TRANSCEND^a^ (10)	US	256	73%	53%	PFS 44% (1y); OS 58% (1y)
**MCL**						
Bre-cel	ZUMA-2^a^ (11)	Global	60	93%	67%	PFS 61% (12m); OS 83% (12m)
**FL**						
Axi-cel	ZUMA-5^a^ (12)	Global	86	94%	79%	NR
**B-ALL**						
Tisa-cel	ELIANA^a^ (13)	Global	75	81%	60%	EFS 50% (12m); OS 76% (12m)
	Pasquini et al. (8)	US & CAN	255	NR	86%	EFS 52% (12m); OS 77% (12m)
Bre-cel	ZUMA-3^a^ (14)	Global	55	71%	56%	Median RFS 11.6m; median OS 18.2m
**MM**						
Ide-cel	KarMMa^a^ (15)	Global	128	73%	33%	Median PFS 8.8m; OS 78% (12m)
Cilta-cel	Cartitude-1^a^ (16, 17)	US	97	97%	67%^b^	PFS 77% (12m); OS 89% (12m)

N, number of patients; ORR, overall response rate; CR, complete response; LBCL, large B-cell lymphoma; Axi-cel, axicabtagene ciloleucel; PFS, progression-free survival; OS, overall survival; y, year; m, month; NR, not reported; Tisa-cel, tisagenlecleucel; RFS, relapse-free survival; EFS, event-free survival; Liso-cel, lisocabtagene maraleuecel; MCL, mantle cell lymphoma; Bre-cel, brexucabtagene autoleucel; FL, follicular lymphoma; B-ALL, B-cell acute lymphoblastic leukemia; MM, multiple myeloma; Ide-cel, idecabtagene vicleucel; Cilta-cel, ciltacabtagene autoleucel.

a pivotal trials.

b stringent CR.

Laboratory optimization mainly focuses on CAR engineering and searching for new targets (20). At the same time, increasing efforts have been made to harness other oncological treatments along with CAR T cell therapy in pursuit of synergistic antitumor effects. The modalities and timing of combination therapies are diverse. After apheresis and before CAR T cell infusion, the combination with bridging therapies and lymphodepletion aims at preparing patients with low tumour burden and creating a favourable microenvironment for CAR T cells. Simultaneously, CAR T cells are produced, and during this process pharmacological agents can be added in culture settings to produce potent T cells for adoptive transfer. Infusion of CAR T cells ensues, and this is when the interactions between CAR T cells and combined agents should be deliberately considered if a concurrent or consolidative treatment is going to be conducted to avoid the impaired fitness of CAR T cells. The main combinatorial strategies include traditional haematological cancer treatment, such as chemotherapy, radiotherapy, haematopoietic stem cell transplantation (HSCT) and small molecule-based targeted therapies. In addition, coupling CAR T cell therapy with other immunotherapies is under intense investigation. Herein, we summarize the available evidence and clinical experience to date on different combination strategies, hoping to provide insight to optimize applications of CAR T cell therapy.

## Challenges of efficacy of CAR T cell therapy in haematological malignancies

**Figure 1:**
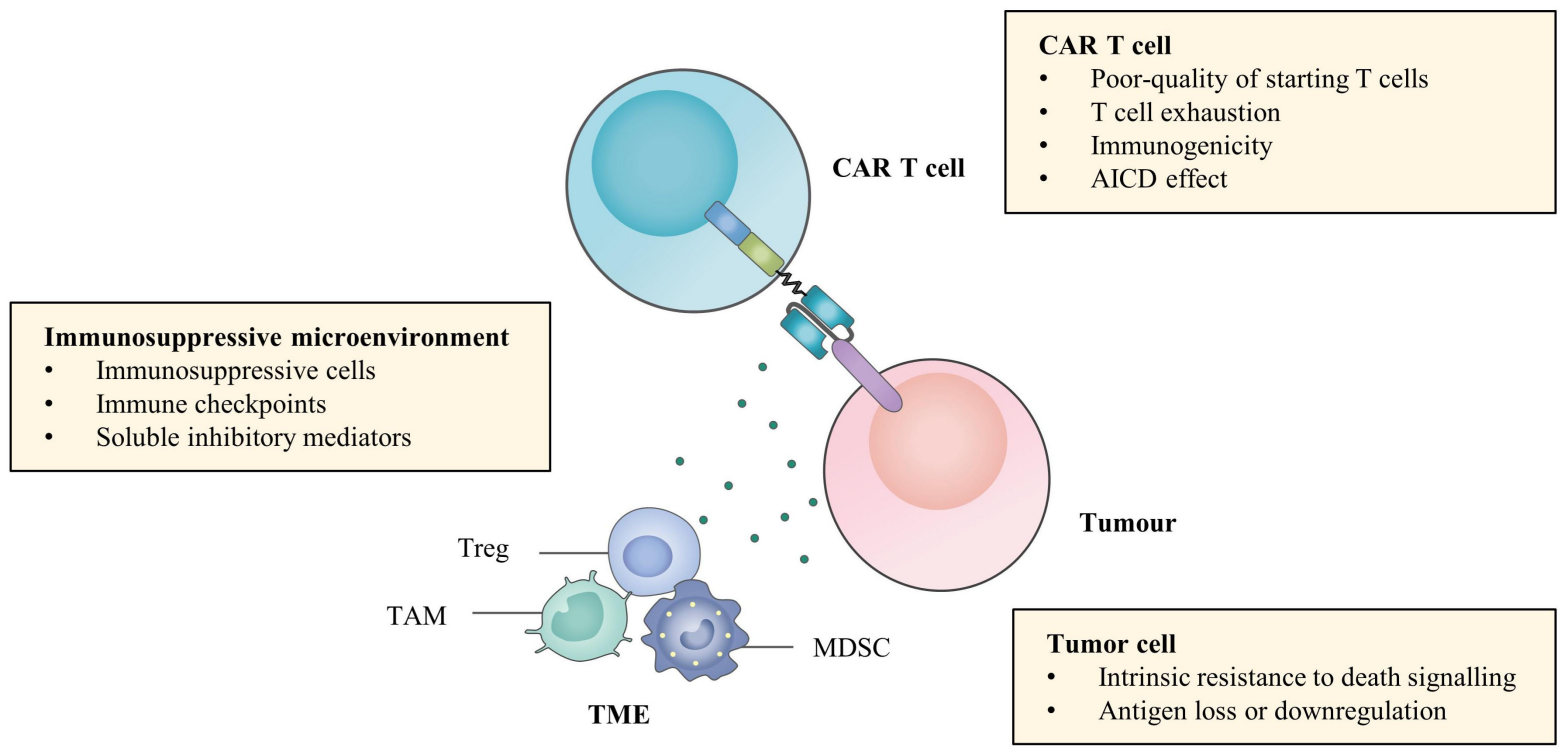
Challenges of efficacy of CAR T cell therapy. Limited efficacy of CAR T cell therapy in haematological malignancies can be attributed to poor persistence of CAR T cells, antigen modulation and intrinsic resistance to death signalling of tumour cells and immunosuppressive microenvironment.

The resistance and relapse of CAR T cell therapy are the result of interactions among CAR T cells, tumour cells and the tumour microenvironment (TME) **(** **Figure 1** **)**. The poor persistence of CAR T cells is the most important reason for antigen-positive relapse. The fitness of T cells collected from patients is likely to be devastated by tumour and frontline treatments, resulting in insufficient CAR T cell quantity and poor quality (21). In addition, sustained antigenic stimulation *in vivo* tends to cause exhaustion of CAR T cells by transcriptomic and epigenetic modulation, whose features include impaired persistence and cytotoxicity, upregulation of inhibitory receptors, metabolic changes and increased apoptosis. CAR molecule can also mediate antigen-independent tonic signalling (22). Another mechanism for less persistent CAR T cells *in vivo* can be immune rejection of the murine-derived single-chain variable fragment (scFv), which is adopted by most clinically available CAR T cell products (23). The activation-induced cell death (AICD) effect also leads to irreversible clearance of CAR T cells (24).

In terms of tumour cells, it is common to modulate antigen expression and escape CAR T cell recognition by various mechanisms. CD19-negative relapse has been studied most widely, and its mechanisms include alternative splicing, immune selection, lineage switching, trogocytosis, epitope masking and methylation silencing (25). MM relapse from B cell maturation antigen (BCMA) CAR T cell therapy occurs through the cleavage of the extracellular domain of BCMA by γ-secretase (GS), a universally expressed intramembrane protease (26). Moreover, biallelic deletion and trogocytosis can also contribute to BCMA antigen escape (27). Sometimes, there is no requirement for complete depletion of antigen expression, and downregulation of the expression of other antigens on haematological malignancies, such as CD22 and CD33, can also achieve resistance (28, 29). On the other hand, tumour cells can resist immune killing *via* intrinsic antiapoptotic pathways. Tumour cells downregulate the expression of death receptors, such as FasL and TNF-related apoptosis-inducing ligand (TRAIL), to become insensitive to the cytotoxicity of CAR T cells (30). They can also increase the expression of endogenous anti-apoptotic proteins such as B cell lymphoma-2 (Bcl-2) and inhibitor of apoptosis proteins (IAPs), resulting in damaged sensitivity to CAR T cell killing (31).

**Figure 2:**
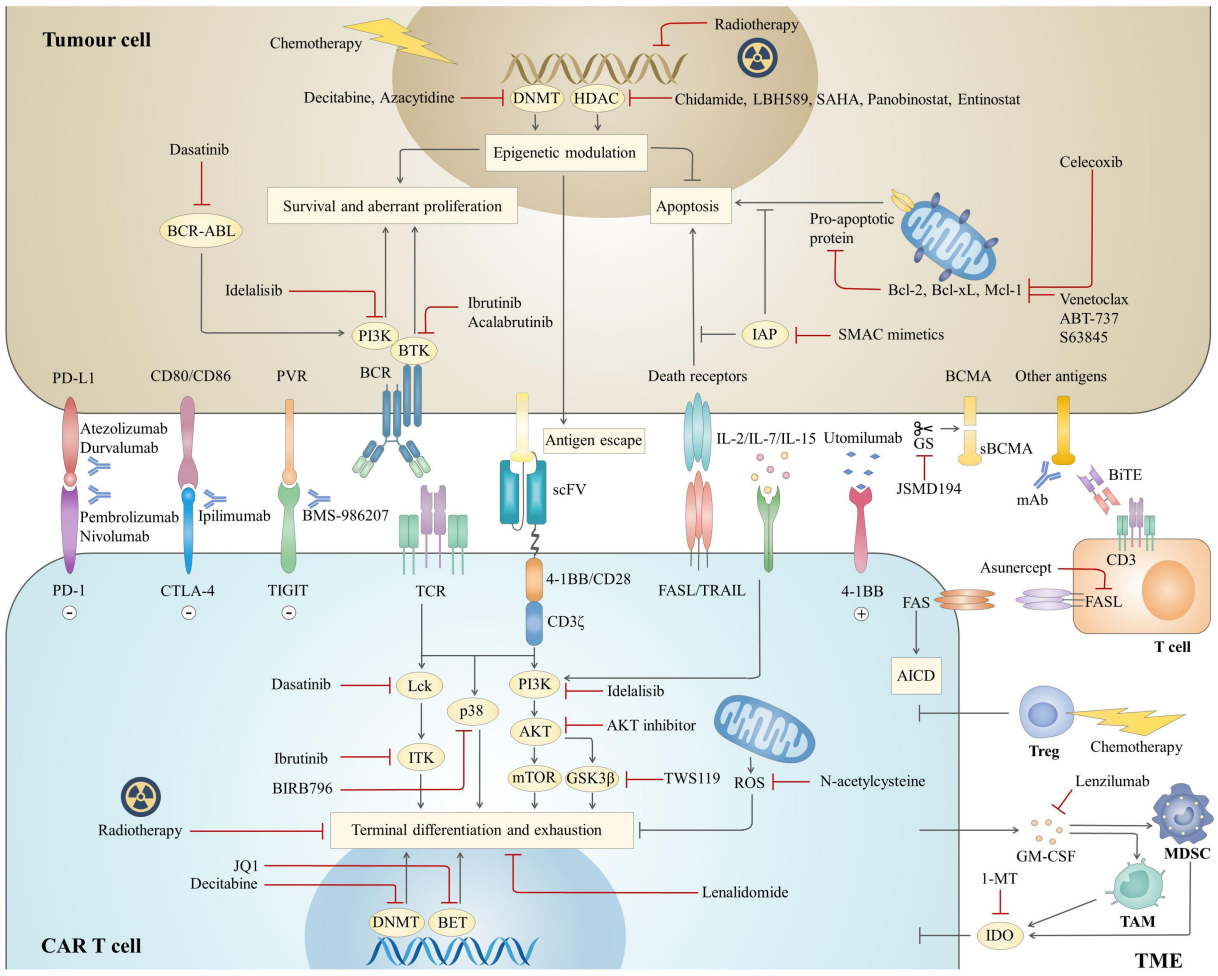
Mechanisms of combination strategies with CAR T cell therapy. The combined agents mainly focus on three aspects to optimize antitumor effects, including tumour cells, CAR T cells and the TME. For CAR T cells, attention is given to targeting different pathways to prevent terminal differentiation and exhaustion. In addition, inhibition of the Fas pathway prevents AICD, the other mechanism to prolong CAR T cell survival. For tumour cells, combined therapies are expected to both directly inhibit tumour survival and boost the cytotoxicity of CAR T cells by sensitizing tumours to apoptotic signalling or upregulating antigen expression. Components of the inhibitory microenvironment are also emerging as promising targets to enhance the effectiveness of CAR T cell therapy. BCR, B cell receptor; sBCMA, soluble B cell maturation antigen; mAb, monoclonal antibody; BiTE, bispecific T cell engager; TCR, T cell receptor; ROS, reactive oxygen species.

The immunosuppressive microenvironment also plays a role in attenuating the potency and persistence of CAR T cells. Although there are still few studies in the field of haematological malignancies, the critical effects of some critical immunosuppressive cells, such as myeloid-derived suppressor cells (MDSCs), tumour-associated macrophages (TAMs) and regulatory T cells (Tregs), immune checkpoints and soluble molecules, such as indoleamine 2,3-dioxygenase (IDO) and adenosine and inhibitory cytokines, on the efficacy of CAR T cell therapy have been gradually elucidated (32). Together, it is the complex interactions among these three components that lead to primary resistance and relapse of CAR T cell therapy and provide targets for combination therapies, aiming at directly eradiating tumour cells as well as improving CAR T cell killing effects **(** **Figure 2** **)**.

## Combination with chemotherapy

Chemotherapy is the mainstay treatment for haematological malignancies and is typically utilized as a preconditioning regimen before CAR T cell infusion. Bridging chemotherapy plays roles in controlling disease progression and reducing tumour burden, and it is strongly associated with improved long-term outcomes and less toxicity **(** **Figure 2** **)** (33). The regimen choices vary depending on patients’ status and prior therapies. On an individual basis, regimens that have been shown to be efficient or have not been used previously can be administered at low intensity to prevent long-term marrow suppression and severe infections, which may delay or exclude patients from CAR T cell infusion. A retrospective study showed that B-ALL patients who received high-intensity myelosuppressive bridging chemotherapy had a higher incidence of severe chemotherapy-related infections than those who received low-intensity myelosuppressive chemotherapy (70% vs. 31%; P<0.001), while there was no difference in overall survival (OS) (33). Shahid et al. gave B-ALL patients with progressive disease (PD) additional cycles of chemotherapy (≥2 cycles) and found that clinical outcomes were not closely associated with tumour burden but rather were associated with the cycles of chemotherapy (34). Those who received more than one cycle of chemotherapy had a higher incidence of severe infections (94% vs. 56%; P=0.019) and lower OS (hazard ratio, HR=3.73, 95% confidence interval, CI, 1.39-9.97; P=0.006) than those who had only one cycle.

Lymphodepletion chemotherapy is generally performed 3-5 days before CAR T cell infusion to create a favourable TME for CAR T cell engraftment, expansion and survival *in vivo* **(** **Figure 2** **)**. The most widely used regimen is cyclophosphamide (Cy) plus fludarabine (Flu). Compared with Cy alone, Cy/Flu significantly improves CAR T cell efficacy (35, 36), possibly because of the properties of Flu in reducing the anti-CAR immune response, increasing the level of homeostatic cytokines and decreasing the expression of the suppressive molecule indoleamine 2,3-dioxygenase (IDO) in the TME (35–37). Generally, Flu is given based on body surface area (BSA), which leads to high interindividual variability of Flu exposure (38, 39). Recent studies indicated that optimizing Flu exposure had a favourable influence on clinical outcomes. In a retrospective analysis, Dekker et al. found that cumulative Flu area under the curve (AUC) _T0-∞_≥14 mg*h/L was strongly associated with improved leukaemia-free survival (LFS) of CD19 CAR T cell therapy (40). Consistently, Fabrizio et al. used a validated population-pharmacokinetic model and found that an estimated Flu AUC≥13.8 mg×h/L was associated with a lower rate of relapse and B cell recovery (41). Prospective clinical trials are warranted for further validation. Alternative regimens are also being explored. Bendamustine is a safer and more tolerable chemotherapy agent with no cross-resistance to Cy (42). In the JULIET trial, bendamustine was used for B-NHL patients who were resistant to Cy (7). In addition, bendamustine plus Flu was found to be more effective than bendamustine or Cy/Flu alone in CD30 CAR T cell therapy for patients with R/R Hodgkin lymphoma (HL) (43, 44). Currently, many efforts to standardize bridging therapy and lymphodepletion regimens are underway. At the same time, the dose of chemotherapy should be optimized, and multiple alternative regimens should be explored to meet individualized needs by different patients and their disease states.

## Combination with radiotherapy

Radiotherapy is a conventional treatment modality for lymphoma with bulky masses. It can also boost the immune response by promoting the recruitment of cytotoxic T cells, reversing T cell exhaustion and releasing tumour antigens, which can theoretically synergize CAR T cell function (45). Additionally, radiotherapy has been proven to sensitize antigen-negative tumour cells to CAR T cell-mediated apoptosis *via* TRAIL **(** **Figure 2** **)** (46). As the bridging therapy of CAR T cell therapy for B-NHL, radiotherapy showed safer and more efficient characteristics than other bridging therapies, such as chemotherapy, monoclonal antibodies, Bruton’s tyrosine kinase (BTK) inhibitors and lenalidomide (47, 48). Although the optimal timing for conducting radiotherapy is unclear, most previous clinical trials administered radiotherapy after apheresis, protecting T cells from potential radiation damage (48, 49). For the field size, bridging radiotherapy is usually delivered focally to the primary tumour and/or specific metastatic sites. However, Pinnx et al. reported that focal radiotherapy excluding active disease has a higher risk of relapse, but those encompassing all sites of lymphoma benefited more (48). Of 8 B-NHL patients who received focal radiotherapy, 17% showed 1-year PFS and 28% OS. Six patients progressed or relapsed, among whom 3 occurred outside the radiotherapy field. For 9 patients who received comprehensive radiotherapy for all lymphoma sites, the 1-year PFS and OS were 57% and 71%, respectively. In terms of B-ALL, radiotherapy is not the standard treatment and is rarely coupled with CAR T cell therapy. However, it is considered a promising strategy for removing extramedullary lesions and increasing the sensitivity of CAR T cell killing (50, 51). Together, radiotherapy is a promising combined treatment regimen and has shown preliminary superiorities. More direct comparisons with other combined therapies are required for further validation, and exploration of the optimal dose and field size of radiation is warranted.

## Combination with HSCT

**Figure 3:**
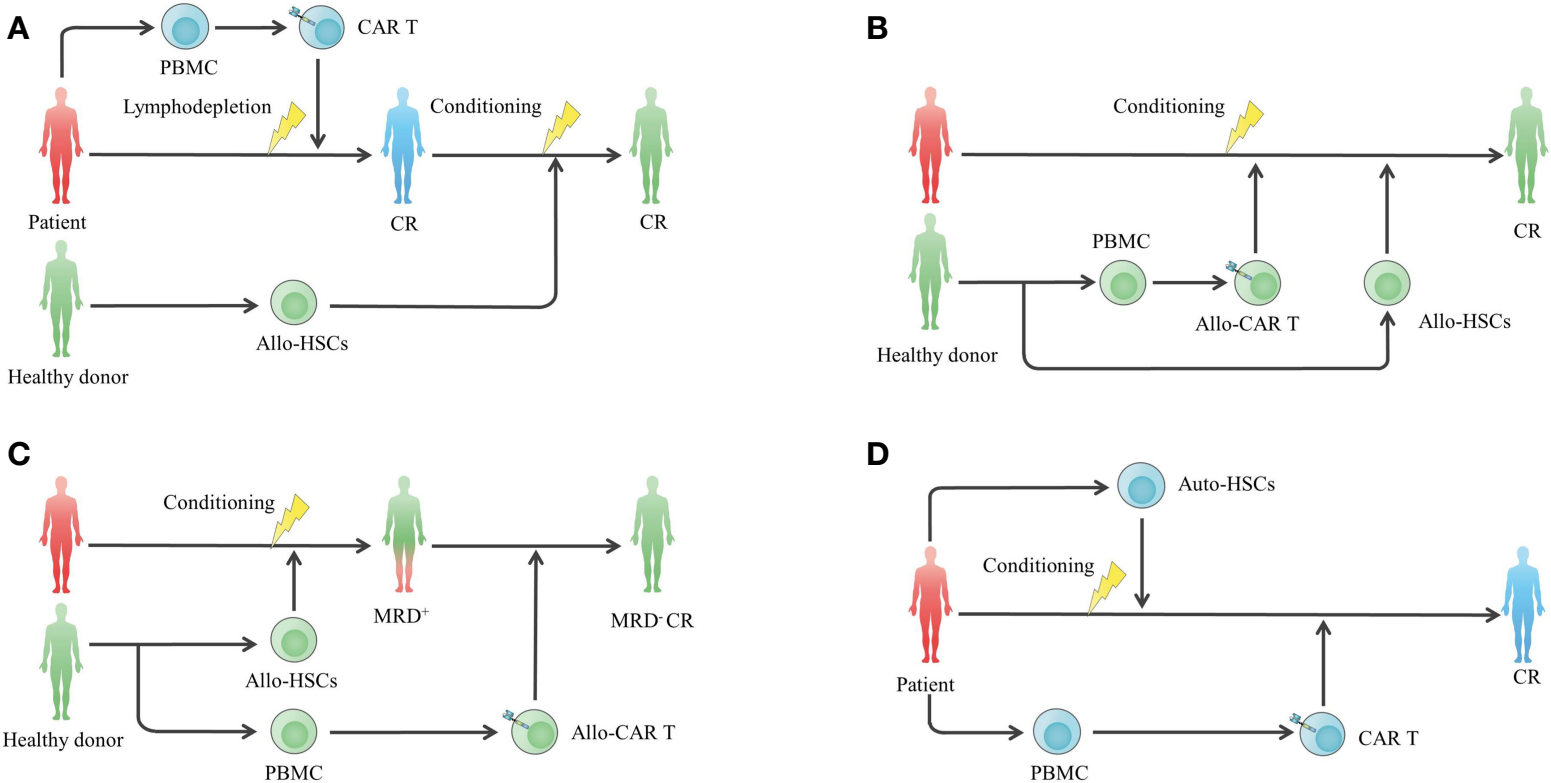
HSCT in combination with CAR T cell therapy to enhance efficacy. **(A)** Standard-of-care CAR T cell therapy following consolidative allo-HSCT. **(B)** Concurrent infusion of allo-CAR T cells and allo-HSCT to enhance antitumor effects and prolong the persistence of allo-CAR T cells without additional gene editing against graft rejection. **(C)** Allo-HSCT following allo-CAR T cell therapy for MRD clearance. **(D)** High-dose chemotherapy conditioning and autologous stem cell transplantation to remodel the immune environment for subsequent CAR T cell therapy. allo-HSC, allogeneic haemopoietic stem cell.

HSCT has been regarded as the only potentially curative treatment in haematological malignancies for a long time, and it has been pursued to utilize this potential treatment to optimize CAR T cell therapy **(** **Figure 3** **)**. Consolidative allogeneic HSCT (allo-HSCT) after CAR T cell therapy is an area of intense research, and it aims to nonselectively eradicate residual tumour cells *via* the graft-versus-leukaemia (GVL) effect, preventing antigen-positive and antigen-negative relapse at the same time. For B-ALL patients, conflicting results exist regarding whether patients can benefit from consolidative allo-HSCT **(** **Table 2** **)**. Recently, a meta-analysis pooled the studies and reported that the OS (HR=0.34, 95% CI, 0.17~0.68, P=0.003) and LFS (HR=0.15, 95% CI, 0.08~0.28, P<0.001) of the bridging group were markedly improved and the relapse rate (HR=0.16, 95% CI, 0.10~0.25, P<0.001) was decreased compared with the nonbridging group (72). The incidence of transplantation-related toxicities did not increase significantly (72). Large-scale randomized controlled trials are warranted to further validate the efficacy and safety of the combinatorial strategy.

**Table 2:** Selective trials for combinations of CAR T cell therapy with HSCT.

Trial	Disease	N	Target	Outcome	Toxicity
**CAR T cell therapy following consolidative allo-HSCT**					
Gu et al. (2021) (52)	B-ALL	30	CD19	OS (2y): allo-HSCT (30): 59%; non-HSCT (26): 23% (HR=0.29; 95%CI: 0.13–0.65; P=0.001) LFS (2y): allo-HSCT: 53%; non-HSCT: 19% (HR=0.20; 95%CI: 0.09–0.45; P<0.001)	CRS: 43%; ICANS: 9% aGVHD: 30%; cGVHD: 50%
Li et al. (2021) (53)	B-ALL	137	CD19/CD22	OS (2y): 74%; LFS (2y): 64%	grade II-IV aGVHD: 42%; cGVHD: 18%
Hu et al. (2021) (54)	B-ALL	52	CD19	OS (2y): 84%; EFS (2y): 76%	CRS: 58%; ICANS: 15% aGVHD: 28%; cGVHD: 35%
Zhao et al. (2020) (55)	B-ALL	55	CD19	OS (2y): allo-HSCT (55): 77%; non-HSCT (67): 36% (HR=0.275; 95%CI: 0.142-0.531; P<0.001) LFS (2y): allo-HSCT: 66%; non-HSCT: 33% (HR=0.244; 95%CI: 0.136~0.437; P<0.0001)	CRS: 93%; ICANS: 75% aGVHD: 64%; cGVHD: 40%
Zhang et al. (2020) (56)	B-ALL	75	CD19	OS (1y): allo-HSCT (75): 79%; non-HSCT (40): 32% (HR=0.048; 95% CI: 0.016-0.148; P<0.0001) LFS (1y): allo-HSCT: 77%; non-HSCT: 12% (HR=35.45; 95%CI: 11.6-108.4; P<0.0001)	CRS: 92%; ICANS: 21%
Park et al. (2018) (57)	B-ALL	17	CD19	OS (2y): allo-HSCT (17): 33%; non-HSCT (36): 45% (P=0.89) EFS (2y): allo-HSCT: 24%; non-HSCT: 27% (P=0.64)	CRS: 85%; ICANS: 43% 6 TRM
Shadman et al. (2019) (58)	B-NHL/CLL	13	CD19	OS (1y): 59% (95% CI: 0.37-0.95)	aGVHD: 85%; cGVHD: 8%
**Allo-HSCT following allo-CAR T cell therapy**					
Zhao et al. (2022) (59)	B-ALL	12	CD19	OS (424.5d): 100%; DFS (424.5d): 65.6%	CRS: 66.7% aGVHD: 0%
Kebriaei et al. (2016) (60)	B-ALL/DLBCL	19	CD19	ORR: 57.9%; CR: 52.6% OS (1y): 63%; PFS (1y): 53%	aGVHD: 15.8%
**Allo-CAR T cell concurrent with allo-HSCT**					
Yang et al. (2021) (61)	B-NHL/ MM	11	CD19/CD20/CD22/BCMA	ORR: 55%; CR: 46%	CRS: 100% aGVHD: 3pts
**CAR T cell therapy following ASCT**					
Wei et al. (2022) (62)	B-NHL	57	CD19+CD22	ORR: 95%; CR: 85%	CRS: 95%; ICANS: 19%
Wang et al. (2022) (63)	B-NHL	21	CD19	ORR: 90%; CR: 71%; OS (3y): 80%; PFS (3y): 80%	CRS: 76%; ICANS: 0%
Cao et al. (2021) (64)	B-NHL	42	CD19+CD22	ORR: 91%; CR: 81% PFS: 83% (2y)	CRS: 96%; ICANS: 21%
Hu et al. (2021) (65)	B-NHL	12	CD22+CD20	ORR: 58%; CR: 58%; OS (9m): 71%; PFS (9m): 67%	CRS: 92%; ICANS: 8%
Wu et al. (2021) (66)	B-NHL	13	CD19+CD22	ORR: 82%; CR: 55%; OS (1y): 83%; PFS (1y): 75%	CRS: 85%; ICANS: 27%
Wang et al. (2020) (67)	B-NHL	14	CD19	ORR: 79%; CR: 43%; OS (1y): 65%; PFS (6m): 64%	CRS: 86%; ICANS: 0%
Sauter et al. (2019) (68)	B-NHL	15	CD19	ORR: 53%; CR: 53%; PFS (2y): 30%	CRS: 40%; ICANS: 67%
Wang et al. (2016) (69)	B-NHL	16	CD19	ORR: 81%; CR: 56%; PFS (1y): 63%	≥grade II CRS: 0%; ICANS: 0%
Garfall et al. (2018) (70)	MM	10	CD19	ORR: 80% (PR: 2; VGPR: 6)	CRS:10% autologous GVHD: 10%
Shi et al. (2018) (71)	MM	9	CD19+BCMA	ORR: 100%; CR: 33.3%	CRS: 100%

CAR, chimeric antigen receptor; HSCT, hemopoietic stem cell transplantation; N: number of patients; B-ALL, B-cell acute lymphoblastic leukemia; Allo-HSCT, allogeneic HSCT; OS, overall survival; EFS, event-free survival; LFS, leukemia-free survival; HR, hazard ratio; CRS, cytokine release syndrome; ICANS, immune effector cell-associated neurotoxicity syndrome; aGVHD, acute graft versus host disease; cGVHD, chronic GVHD; B-NHL, B-cell non-Hodgkin lymphoma; CLL, chronic lymphocytic leukemia; DFS, disease-free survival; pts, patients; DLBCL, diffuse large B cell lymphoma; PFS, progression-free survival; d, day; MM, multiple myeloma; ORR, overall response rate; CR, complete response; PR, partial response; VGPR, very good partial response; TRM, transplant-related mortality.

To date, decisions regarding whether to consolidate allo-HSCT should be made individually, considering the costs and risk of fatal transplantation-associated toxicities. Patients with a high tumour burden (≥5%) before CAR T cell infusion or poor prognostic factors, including high-risk genetic mutations and complex karyotypes, are more likely to benefit from consolidative allo-HSCT because of their high relapse risk (73, 74). Of note, it is believed that MLL-rearranged, BCR/ABL1 and TCF3-ZNF384 are associated with CD19-negative relapse *via* lineage switching to the myeloid lineage (75, 76), and patients with these markers are more likely to benefit from allo-HSCT to eliminate the origin of CD19-negative relapse. In addition, the detection of leukaemic sequences in bone marrow, CAR T cell loss and B cell recovery can predict a higher probability of relapse, facilitating timely allo-HSCT consolidation (76). In terms of timing for bridging allo-HSCT, early transplantation is likely to be a more effective and safer choice, while a ≥80-day interval may cause decreased OS and increased toxicities (58, 77). With regard to B-NHL and MM, few attempts to administer consolidative allo-HSCT have been made, and the efficacy is unclear.

When combining allo-HSCT with the same donor-derived CAR T cells, which are suitable for heavily treated patients who cannot harvest adequate T cells for autologous CAR T cell manufacturing (78), the haematopoietic remodelling function of allo-HSCT can be utilized to help graft allogeneic CAR T cells to avoid additional gene editing. Seven R/R B-NHL and 2 R/R MM patients who relapsed from autologous CAR T cells received allogeneic CAR T cells in combination with busulfan, a Flu-based conditioning regimen. Seven days later, allo-HSCT was performed in combination with graft-versus-host disease (GVHD) prophylaxis (79). The overall response rate (ORR) and complete remission (CR) were 54.5% and 45.5%, respectively, and the OS was 63.6% in a median follow-up of 171 days. A total of 81.8% of patients had persistent allogeneic CAR T cell survival, and the longest survival lasted up to 239 days after allo-HSCT. Adverse events included controllable cytokine release syndrome (CRS) and mild acute GVHD, preliminarily showing the safety and efficacy of the combined approach. Despite this finding, it is a challenge to manage adverse events caused by donor cell infusion because the application of GVHD prophylaxis is likely to cause compromised CAR T cell persistence (80–82). More clinical data are required to find a balance between safety and long-term effectiveness. Donor-derived CAR T cells can also be used as a consolidative regimen after allo-HSCT, and it is more effective than donor lymphocyte infusion in minimal residual disease (MRD) clearance (59, 60). In addition, the safety of CAR T cell infusion after allo-HSCT is likely to be improved because of the reduced tumour burden by HSCT before CAR T cell infusion (59, 60).

Autologous haematopoietic stem cell transplantation (ASCT) is also a standard of care for haematological tumours and can be utilized to enhance adoptive T cell functions by reducing tumour cells and remodelling the immune environment for T cell expansion (68, 83). The feasibility of CAR T cell therapy shortly following high-dose chemotherapy and ASCT (HDT-ASCT) has been confirmed in several clinical studies **(** **Table 2** **)**. Wei et al. compared CAR T cell infusion after ASCT and CAR T cells alone and demonstrated that the best ORR, CR rate and long-term outcomes improved significantly in the combination group (62). In another study comparing CAR T cell infusion after ASCT and ASCT alone, the combination group showed higher CR rate (71% vs. 33%; p=0.003) and 3-year PFS (80% vs. 44%; p=0.036) than the ASCT group, and demonstrated lower 3 year relapse/progression rate (15% vs. 56%; p=0.015) (63). Incidences of adverse events differ from studies and were manageable in most cases **(** **Table 2** **)**. However, Sauter et al. highlighted aggravated immune effector cell-associated neurotoxicity syndrome (ICANS) in their study. Sixty-seven percent of poor-risk R/R B-NHL patients who underwent BEAM conditioning (carmustine, etoposide, cytarabine, and melphalan) and ASCT following CD19-CD28 CAR T cell infusion experienced severe ICANS, possibly resulting from wide adoption of pegylated G-CSF post HDT-ASCT (68). Moreover, a study explored prolonging the interval between ASCT and CAR T cell therapy for adequate haematological recovery after ASCT (84). It showed that CAR T cells could further induce CR without aggravated toxicity for those in whom CR was not induced by ASCT. The feasibility of this strategy is further being explored (NCT04923789). In MM treatment, busulfan and Cy (BUCY) conditioning and ASCT followed by infusion of CD19 CAR T cells or their coupling with B cell maturation antigen (BCMA) CAR T cells is also a promising regimen (66, 71).

The various combined modalities between HSCT and CAR T cells provide more treatment options for haematological malignancies, among which whether to consolidate allo-HSCT after CAR T cell therapy is the most researched. High-quality randomized controlled trials to guide clinical decisions are expected. ASCT bridging to CAR T cell therapy is also likely to exert potent anti-tumour efficacy, while further validation and comparisons with other bridging therapies before CAR T cell infusion are warranted.

## Combination with targeted therapy

### BTK inhibitors

Ibrutinib, the first BTK inhibitor, regulates B cell receptor signalling and the development of B cells and has been approved for chronic lymphocytic leukaemia (CLL) and mantle cell lymphoma (MCL) (85). It also plays an immunoregulatory role *via* the inhibition of interleukin-2 inducible T cell kinase (ITK), which may have a positive effect on the expansion and function of CAR T cells (86).

Patients with a history of BTK inhibitor exposure are likely to benefit more from robust CAR T cells (87, 88). Furthermore, pilot studies confirmed the feasibility of BTK inhibitor administration prior to or concurrent with CAR T cell infusion. Nineteen patients with ibrutinib-refractory CLL were scheduled for ibrutinib infusion from 2 weeks before leukapheresis to more than 3 months after CAR T cell infusion. The ORR was 83%, and 61% achieved an MRD-negative marrow response. The estimated 1-year OS and PFS rates were 86% and 59%, respectively. The toxicities included 14 cases of mild CRS and 5 cases of grade 3 ICANS (89). Similarly, in a phase II clinical trial, 19 patients with CLL were infused with CAR T cells along with a continuous infusion of ibrutinib. The three-month CR was 44%, and 72% of patients were MRD-negative at the 1-year follow-up. The estimated 48-month OS and PFS were 84% and 70%, respectively. Eighteen patients developed CRS, including 3 cases of severe CRS, and 5 patients developed ICANS, including 1 case of grade 4 ICANS (90). Apart from *in vivo* administration of ibrutinib, adding ibrutinib into an *ex vivo* culture system can also increase the number of CAR T cells and decrease the expression of exhaustion markers (91).

Ibrutinib combination therapy has also been applied in B-NHL. Liu et al. reported that 7 MCL/follicular lymphoma patients who did not achieve CR by CD19 CAR T cell therapy received ibrutinib as salvage treatment for 7-16 months, followed by a second CAR T cell infusion. A significant improvement in the *in vivo* expansion of CAR T cells was observed (median peak of CAR T cells in CD3+ T cells 32.11% ± 13.28% vs. 20.34% ± 10.70%), and 6 patients achieved CR (92). In ZUMA-2, the analysis of CAR T cells and peripheral blood mononuclear cells (PBMCs) collected from MCL patients 7 days after infusion showed that compared with acalabrutinib-exposed CAR T cells, ibrutinib-exposed CAR T cells had a T helper cell (Th)1 predominant phenotype and were more likely to shift from a central memory phenotype (Tscm) to an effector memory phenotype (Tem) for the enhancement of function, possibly because of additional targeting of ITK by ibrutinib compared with acalabrutinib (93). With regard to the safety of the combination therapy, ITK inhibition by ibrutinib can prohibit cytokine hyperproduction and reduce the incidence of CRS (87, 89). However, ibrutinib may cause cardiac toxicity, and fatal arrhythmia has been reported in combination treatment (89, 94), emphasizing the need for continuous monitoring.

Acalabrutinib is a second-generation BTK inhibitor. Although it does not target ITK, it has higher selectivity and may cause fewer adverse events (95). In a B-ALL mouse model, the combination of acalabrutinib and CD19 CAR T cells prolonged the durability of CAR T cells *in vivo* and improved the survival of mice (96). Clinical trials of combined acalabrutinib with CAR T cells for B cell lymphoma have been launched (NCT04257578; NCT04484012).

### PI3K-Akt-mTOR pathway inhibitors

The phosphatidylinositol-3-kinase (PI3K)-Akt-mTOR pathway is a popular therapeutic target for various tumour cells (97). It also promotes T cell proliferation and differentiation by regulating metabolism and transcription (98). *Ex vivo* PI3K blockade promotes Tscm, central memory T cell (Tcm) and naïve T cell (Tn) development without perturbing expansion; decreases exhaustion marker expression; and increases the ratio of CD8/CD4 T cells, facilitating *in vivo* expansion and cytotoxicity (98, 99). The CRB-402 (NCT03274219) trial is evaluating BCMA CAR T cells pretreated with the PI3K inhibitor bb007 *ex vivo* for R/R MM. The updated results showed a prolonged duration of response (DOR), and the median DOR of 72 patients was 17 months (100). Inhibition of the downstream Akt during the CAR T cell manufacturing process can also harvest products with a higher percentage of early memory phenotype (101, 102). In addition, the mTORC1 inhibitor rapamycin could upregulate CXCR4 expression on CAR T cells for bone marrow migration and inhibit terminal effector T cell differentiation but with the risk of attenuating T cell proliferation *ex vivo* (99, 103).

### Tyrosine kinase inhibitor

Dasatinib, a commercial tyrosine kinase inhibitor (TKI), reversibly inhibits phosphorylation of proximal TCR and CAR signalling kinases, preventing activation and differentiation induced by toxic CAR signalling and reversing T cells that have already been exhausted by epigenetic remodelling (104, 105). This mechanism facilitates *ex vivo* manufacturing to turn off CAR signalling and produce high-quality CAR T cells (104, 105). On the other hand, when introducing dasatinib into *in vivo* administration, long-term resting CAR T cells may increase the risk of tumour progression. Therefore, an intermittent and pulsed administration regimen is a potential strategy (105). Additionally, the rapid and reversed inhibitory profile of dasatinib indicates that it is an ideal agent for attenuating CRS and making CAR T cell therapy more controllable (106).

### γ-secretase inhibitor

GS inhibitor (GSI), clinically used for cancers and Alzheimer’s disease (107), can effectively prohibit the cleavage of BCMA on MM cells by GS (26, 108). MM patients who received GSI showed increases in BCMA density on myeloma cells and decreases in soluble BCMA. As a result, the ORR of 6 patients, including those who had failed prior BCMA targeted therapy, was 100% (5 very good partial remission (VGPR); 1 PR) (108). Long-term follow-up is warranted. Furthermore, epigenetic modulation by all-trans retinoic acid (ATRA) could be synchronized with GSI to further increase BCMA density (109).

### Bcl-2 inhibitors

Bcl-2 family members are key regulators of the intrinsic apoptosis pathway (110). Venetoclax, an inhibitor of the antiapoptotic protein Bcl-2, has been approved for CLL and acute myeloid leukaemia (AML) and clinically confirmed to be efficient for B-ALL (111, 112). It was demonstrated to promote the release of proapoptotic proteins and increase CD19 expression by priming tumour cells with venetoclax, making tumours more sensitive to CAR T cell killing (113). In contrast, concurrent or post CAR T cell treatment is likely to impair the repetitive killing effect of CAR T cells by directly inducing apoptosis and inhibiting proliferation (113). However, there is an opposite argument that concurrent administration of venetoclax selectively affects CAR T cells with different immunophenotypes, supporting stem-like CD8+ T cell proliferation and decreasing Tregs. This conversion results in synergy with CAR T cell therapy (114). Further investigations on CAR T cell biology under exposure to venetoclax are warranted. Other antiapoptotic Bcl-2 family inhibitors, such as ABT-737 and myeloid leukaemia 1 (Mcl-1) inhibitor S63845, can also presensitize B cell malignancies to CAR T cell therapy (113, 115).

### Targeting cytokines

During *ex vivo* CAR T cell production, cytokines are usually involved in potentiating expansion. IL-2 is the most common cytokine but is likely to induce CAR T cell terminal differentiation and increase the Treg proportion (116, 117). In contrast, other γ-chain cytokines IL-7 and IL-15 can preserve the proliferative capability of CAR T cells without hampering *ex vivo* expansion, which contributes to better *in vivo* persistence and antitumor effects (117, 118). In addition, IL-21 can preserve a less differentiated CAR T cell phenotype with other strengths, such as enhancing transfection efficiency and cytolytic activities when combined with other γ-chain cytokines, while IL-21 alone may not be able to support sufficient CAR T cell expansion (117, 119, 120). Other cytokine family members, such as transforming growth factor-β (TGF-β), have also been proven to induce early memory T cell subsets *ex vivo* and subsequently potentiate the function of BCMA CAR T cells *in vivo* without a suppressive effect on T cell expansion (121).

After CAR T cell infusion, cytokines can also be administered to assist expansion and persistence. Fourth-generation CAR T cells are genetically engineered to secrete cytokines. By comparison, pharmacological grade cytokines may be more tunable and convenient as adjuvants. Administration of NKTR-255, a polymer-conjugated human IL-15, in combination with CD19 CAR T cells increased CAR T cell numbers, cytotoxicity and survival in a murine lymphoma model (122). R/R B-NHL and MM patients who had progressed or relapsed after CAR T cell therapy were administered with NKTR-255, and re-expansion of CAR T cells and other CD8+ T cells was subsequently observed (123). Clinical trials for consolidative NKTR-255 following CAR T cell therapy are ongoing (NCT03233854; NCT05359211). In addition, NT-I7, a long-acting human IL-7, could be used as a consolidative regimen *in vivo* to enhance the expansion, persistence and antitumor activities of CAR T cells in lymphoma and leukaemia mouse models (124). A clinical trial is currently ongoing to test the efficacy of NT-I7 administered 21 days after CD19 CAR T cell infusion in lymphoma patients (NCT05075603). Other immune stimulatory cytokines, such as IL-18 and IL-12, can also enhance the killing effects of CAR T cells (125, 126). Their coupling with CAR T cells in haematological malignancies deserves future exploration.

### Epigenetic modulators

Epigenetic modulators are emerging oncological treatment agents that target aberrant epigenetic regulation and inhibit the occurrence and development of tumour cells (127). Meanwhile, epigenomic modulation results in upregulation of tumour antigen expression and improved survival and cytotoxicity of T cells (128), providing rationales for combination strategies with CAR T cell therapy.

Decitabine (DAC), a DNA methyltransferase inhibitor (DNMTi), is commonly used in myelodysplastic syndromes and AML (129). Combining DAC with CD19 CAR T cell therapy can effectively enhance antitumor effects by upregulating CD19 expression on tumour cells and reversing CAR T cell exhaustion (130, 131). A clinical trial of DAC-primed tandem CD19/CD20 CAR T cells for R/R B-NHL is ongoing (NCT04697940). Azacitidine (AZA), another FDA-approved DNMTi, can increase the antigen density of CD70 or CD123 in AML, potentiating CAR T cell targeting (132, 133). Existing data are mainly based on tumours pretreated with AZA, and no attempt to directly expose CAR T cells to AZA has been made because there is a concern that AZA promotes Tregs (134). However, the influence of AZA on CAR T cells may be positive and worth further investigation.

Histone deacetylase inhibitor (HDACi), another popular type of epigenetic modulator, such as chidamide, has been proven to effectively upregulate CD22 on B cell malignancies (135). HDACi can also upregulate tumour pro-apoptotic genes, sensitizing tumour cells to CAR T cell-mediated killing (136). Moreover, the bromodomain and extraterminal domain (BET) family member BRD4 contributes to upregulating the expression of the transcription factor BATF and promoting Tem differentiation (137). The BRD4 inhibitor JQ1 reversed differentiation and enhanced CAR T cell persistence and antitumor effects in B-ALL mouse models (138). The interactions between c-Myc and the epigenetic regulator canonical BRG1/BRM-associated factor (cBAF) promote the remodelling of the chromosomal landscape and the accessibility of T cell differentiation-related gene regions, thereby driving the differentiation of T cells towards effector T cells. Treatment with BRD-K98645985 (BD98), a probable BAF inhibitor, maintained the memory-like phenotype of T cells *in vivo* and enhanced the antitumor function and persistence of CAR T cells in osteosarcoma and glioma models (139). In summary, epigenetic modifications are promising strategies to address resistance from the perspectives of both CAR T cells and tumour cells. Despite compelling preclinical evidence, the efficacy of the combination of epigenomic agents and CAR T cell therapy needs to be tested in clinical trials.

### Other targeted therapies

With profound mechanisms of resistance and relapse of CAR T cell therapy, more potential targets and corresponding pharmacological treatments are emerging **(** **Table 3** **)**. Preventing and reversing CAR T cell exhaustion is one of the most intense study fields. Along with the strategies mentioned above, such as targeting the PI3K-Akt pathway and TKI, another T cell expansion and differentiation-associated kinase glycogen synthase kinase-3β (GSK-3β) was found to be inhibited by TWS119 *ex vivo* for memory maintenance (165–167). In addition, metabolic changes are a characteristic of CAR T cell exhaustion with impaired antioxidant capacity. Scavenging of reactive oxygen species by the antioxidant N-acetylcysteine can effectively promote Tscm formation and expansion *ex vivo* for better product manufacturing (168). Ideal phenotypes of cytotoxic T cells *in vivo* are believed to comprise expansion, differentiation, oxidative and genomic stress, and CRISPR-cas9-based genetic screening of TCR-based kinases identified Mapk14(p38) as a central regulator of those determining factors (141). Targeting it with BIRB796 *ex vivo* significantly enhanced the antitumor effects of CAR T cells *in vivo*. On the other hand, overactivation of T cells triggers Fas-mediated AICD, which can be abrogated by asunercept, a FasL inhibitor, subsequently increasing the number of CD19 CAR T cells and enhancing antitumor killing effects (142). However, this strategy may attenuate Fas-mediated cytotoxicity against tumour cells or trigger autoimmune diseases (169).

**Table 3:** Rationales for the combination of CAR T cell therapy with targeted therapies and other immunotherapies.

Category	Mechanisms	Agents	Status	Regimen
BTK inhibitor	-Inhibit BCR and development of tumour cells -Ibrutinib also inhibits ITK to promote expansion and cytotoxicity of CAR T	Ibrutinib	Published clinical trials (89–91)	Prior to, concurrent with and after CAR T *Ex vivo*
		Acalabrutinib	Ongoing (NCT04257578; NCT04484012)	Prior to, concurrent with and after CAR T
		Zanubrutinib	Ongoing (NCT05202782)	Prior to, concurrent with and after CAR T
		Bb007	Ongoing (NCT03274219)	*Ex vivo*
PI3K inhibitor	-Maintain less differentiated T cells and promote expansion -Decrease exhaustion marker expression -Increase ratio of CD8/CD4 -Target PI3K pathway in tumour cells	Duvelisib	Ongoing (NCT05044039; NCT04890236)	Prior to, concurrent with and after CAR T *Ex vivo*
		Idelalisib	Preclinical (140)	
		
		LY294002	Preclinical (99)	*Ex vivo*
AKT inhibitor	-Maintain less differentiated T cells	AKTi-1/2	Preclinical (101, 102)	*Ex vivo*
TKI	-Suppress tonic CAR signal reversibly -Reverse exhaustion by epigenetic remodelling	Dasatinib	Ongoing (NCT04603872)	Pulsed administration after CAR T infusion *Ex vivo*
GSK-3β inhibitor	-Maintain less differentiated T cells and promote expansion	TWS119	Ongoing (NCT01087294)	*Ex vivo*
P38 inhibitor	-Play central regulatory role in T cells expansion, differentiate, oxidative and genomic stress	BIRB796	Preclinical (141)	*Ex vivo*
Antioxidant	-Scavenge ROS and promote stem memory T cells formation	N-acetylcysteine	Ongoing (NCT05081479)	Prior to, concurrent with and after CAR T *Ex vivo*
FasL blockade	-Prevent AICD in CAR T	Asunercept	Preclinical (142)	After CAR T
Cytokines	-Promote T cells expansion, memory maintenance and cytotoxic potential	IL-15 and IL-7	Ongoing (NCT02652910; NCT02992834)	*Ex vivo*
		NKTR-255	Ongoing (NCT03233854; NCT05359211)	Concurrent with and after CAR T
		NT-I7	Ongoing (NCT05075603)	After CAR T
		IL-15 and IL-21	Preclinical (120)	Concurrent with and after CAR T
GSI	-Inhibit cleavage and downregulation of BCMA	JSMD194	Ongoing (NCT04855136; NCT03502577)	Concurrent with and after BCMA CAR T
PKC inhibitor	-Upregulate CD22 expression	Bryostatin 1	Preclinical (29, 143)	Prior to, concurrent with and after CD22 CAR T
Bcl-2 inhibitor	-Sensitize tumour cells by inhibiting anti-apoptotic Bcl-2 family proteins including Bcl-2, Mcl-1 and Bcl-xL	Venetoclax	Published clinical trial (144); Ongoing (NCT04640909)	Prior to CAR T
		ABT-737	Preclinical (115)	Prior to and concurrent with CAR T
		S63845	Preclinical (113)	Prior to CAR T
COX-2 inhibitor	-Sensitize tumour cells to CAR T killing	Celecoxib	Preclinical (136)	Prior to CAR T
SMAC mimic	-Inhibit IAPs and sensitize tumour cells to CAR T killing	Birinapant; AT-406; LCL-161	Preclinical (145)	Prior to, concurrent with and after CAR T
DNMT inhibitor	-Inhibit abnormal DNA methylation in tumour cells -Upregulate tumour antigen expression -Reverse CAR T exhaustion	Decitabine	Ongoing (NCT04697940; NCT04850560; NCT04553393)	Prior to, concurrent with and after CAR T *Ex vivo*
		Azacitidine	Preclinical (132, 133)	
HDAC inhibitor	-Induce apoptosis of tumour cells -Upregulate tumour antigen expression -Sensitive tumour cells to CAR T killing	Chidamide LBH589; SAHA; Panobinostat; entinostat	Ongoing (NCT05370547; NCT04337606) Preclinical (136)	Prior to CAR T Prior to CAR T
BET inhibition	-Inhibit BRD4 to reverse differentiation of CAR T	JQ1	Preclinical (138)	After CAR T
IDO inhibitor	-Target immunosuppressive metabolites in TME	1-MT	Preclinical (37)	Prior to, concurrent with and after CAR T
GM-CSF inhibitor	-Inhibit immunosuppressive cells	Lenzilumab	Ongoing (NCT04314843)	After CAR T
Immuno-modulator	-Enhance cytotoxicity -Maintain memory phenotype -Skew Th2 towards Th1 -Increase immune synapse	Lenalidomide	Published clinical trials (146–148) Ongoing (NCT03070327; NCT05032820; NCT04133636)	Prior to, concurrent with and after CAR T
		CC-122; CD-220; CC-99282	Ongoing (NCT03310619)	Prior to, concurrent with and after CAR T
Anti-PD-1 mAb	-Block the inhibitory molecule PD-1	Pembrolizumab	Published clinical trials (149–151); Ongoing (NCT03287817; NCT02935257)	Prior and after CAR T
		Nivolumab	Published clinical trials (152); Ongoing (NCT05385263; NCT05352828; NCT05310591; NCT03310619)	Prior to, concurrent with and after CAR T
		Tislelizumab	Ongoing (NCT04381741; NCT04539444)	Concurrent with and after CAR T
Anti-PD-L1 mAb	-Block the inhibitory molecule PD-L1	Atezolizumab	Published clinical trial (153);	After CAR T
		Durvalumab	Published clinical trial (154, 155); Ongoing (NCT03310619)	Prior to, concurrent with and after CAR T
Anti-CTLA-4 mAb	-Block the inhibitory molecule CTLA-4	Ipilimumab	Ongoing (NCT00586391)	After CAR T
Anti-TIGIT mAb	-Block the inhibitory molecule TIGIT	BMS-986207	Preclinical (156)	After CAR T
Monoclonal antibody	-Induce tumour death by ADCC and CDC -Target immunosuppressive cells in TME by CD38 mAb	Rituximab	Published clinical trial (47); Ongoing (NCT04002401)	Prior to, concurrent with and after CAR T for B-NHL
		Obinutuzumab	Published clinical trial (47); Ongoing (NCT04889716)	Prior to and concurrent with CAR T for B-NHL
		Daratumumab	Published clinical trial (157)	Prior to CAR T for MM
ADC	-Bind to tumour surface antigen and release cytotoxic agents	Polatuzumab vedotin	Published clinical trial (158); Ongoing (NCT05260957)	Prior to CAR T for B-NHL
		Inotuzumab ozogamicin	Published clinical trial (159)	Prior to CAR T for B-ALL
		Brentuximab vedotin	Published clinical trial (10)	Prior to CAR T for B-NHL
BiTE	-Bind to T cells and tumour cells simultaneously to further trigger anti-tumour effect of T cells	Mosunetuzumab	Published clinical trial (160) Ongoing (NCT04889716, NCT05260957)	Prior to and after CAR T for B-NHL
		Glofitamab	Published clinical trial (161); Ongoing (NCT04889716)	Prior to and after CAR T for B-NHL
		Blinatumomab	Published clinical trial (162–164)	Prior to CAR T for B-ALL

BTK, Bruton’s tyrosine kinase; BCR, B cell receptor; CAR, Chimeric antigen receptor; PI3K, Phosphatidylinositol-3-kinase; TKI, tyrosine kinase inhibitor; GSK-3β, glycogen synthase kinase-3β; ROS, reactive oxygen species; AICD, activation-induced cell death; GSI, γ-secretase inhibitor; BCMA, B-cell maturation antigen; PKC, protein kinase C; Bcl-2, B-cell lymphoma-2; Mcl-1, myeloid leukaemia 1; COX-2, cyclooxygenase-2; SMAC, second mitochondria-derived activator of caspases; IAPs, inhibitor of apoptosis family of proteins; DNMT, DNA methyltransferase inhibitor; HDAC, Histone deacetylase; BET, bromodomain and extraterminal domain; IDO, indoleamine 2,3-dioxygenase; 1-MT, 1-methyl-d-tryptophan; TME, tumour microenvironment; GM-CSF, granulocyte-macrophage colony-stimulating factor; PD-1, programmed-cell-death-1; mAb, monoclonal antibody; PD-L1, PD-ligand 1; CTLA-4, Cytotoxic T lymphocyte-associated antigen-4; TIGIT, T-cell immunoreceptor with immunoglobulin and immunoreceptor tyrosine-based inhibitory motif domain; Th, T helper; CDC, complement-dependent cytotoxicity; ADCC, antibody-dependent T-cell-mediated cytotoxicity; ADC, antibody-drug conjugate; BiTE, bispecific T-cell engager.

From the perspective of tumour cells, antigen loss or downregulation is the trickiest limitation. In addition to epigenetic modulators and GSI, another small molecule drug, protein kinase C inhibitor bryostatin I, can stabilize CD22 in B-ALL and B-NHL and improve CD22 CAR T cell functionality and durability of response (29). Furthermore, there are some intrinsic resistance mechanisms in tumour cells *via* abnormal interactions between anti-apoptotic and pro-apoptotic molecules. Through high-throughput coculture drug sensitivity screens of more than 500 oncological drugs, the apoptosis-regulating drug SMAC mimic was identified to enhance CD19 CAR T cell toxicity (145). Complementary CRISPR screening identified that SMAC mimics play synergetic roles by inhibiting the IAPs and sensitize leukaemia and lymphoma to death receptor signalling (145). In addition, celecoxib, a well-known cyclooxygenase-2 (COX-2) inhibitor, also contributes to the upregulation of apoptotic proteins, thereby restoring the sensitivity of B-NHL cells to CD19 CAR T cells (136). However, it may unselectively inhibit CAR T cells and increase the risk of CAR T cell apoptosis (79).

Targeting the immunosuppressive TME is another direction for developing combined regimens. Metabolites in the TME, such as IDO and adenosine, are a promising direction. The combination of the IDO inhibitor 1-methyl-tryptophan and CD19 CAR T cells showed improved tumour control in lymphoma models (37). In addition, anti-T cell responses mediated by adenosine can be mitigated by pharmacological blockage of the adenosine 2A receptor (170, 171). Interestingly, GM-CSF inhibitors were initially used to alleviate CRS and ICANS, but they also showed an inhibitory profile towards the proliferation and migration of immunosuppressive cells, consequently enhancing the antitumor effect of CD19 CAR T cells (172).

## Combination with immunotherapy

### Lenalidomide

Lenalidomide, the primary treatment modality for MM, exerts direct antitumor effects as well as immunomodulatory effects. When combined with CAR T cell therapy, it plays a stimulatory role on CAR T cells by enhancing cytotoxicity, maintaining memory phenotype, skewing Th2 towards Th1 and increasing immune synapses (173, 174). In murine models, lenalidomide was further proven to enhance the antitumor effects of CAR T cells and prolong mouse survival significantly (173, 174).

Clinically, 10 newly diagnosed MM patients received sequential CD19 and BCMA CAR T cell treatment after ASCT, followed by lenalidomide maintenance until relapse. Ninety percent of patients achieved stringent CR, and 10% of patients achieved CR. During a median follow-up of 42 months, the median PFS was not reached, and 70% of patients maintained MRD negativity for more than 2 years (148). In addition, R/R MM patients who did not respond to CAR T cell therapy alone are also likely to improve outcomes by CAR T cells in cooperation with lenalidomide (147). A phase I clinical trial is ongoing (NCT03070327).

The combination of lenalidomide and CAR T cells is also viable in B-NHL (146). PiggyBac-produced CD19 CAR T cells induced CR in a triple-hit R/R DLBCL patient with the TP53 mutation. Then, oral lenalidomide was given from the fourth month after CAR T cell infusion, and CAR copies were still detectable 9 months post-infusion. Although the patient stopped lenalidomide after one cycle because of side effects, including rash, itching and joint pain, he maintained CR for more than 2 years, and his OS was over 3 years (146). ZUMA-14 is exploring the clinical efficacy of the combination of axicabtagene ciloleucel and lenalidomide (NCT04002401).

### Immune checkpoint inhibitors

Immune checkpoint inhibitors have been widely used in cancer treatment, among which programmed-cell-death-1 (PD-1)/PD-ligand 1 (PD-L1) are the most significant targets. Combining CAR T cell therapy with PD-1/PD-L1 inhibitors, further unleashing efficacy in haematological tumours, is an attractive strategy.

Pembrolizumab, an anti-PD-1 mAb, was administered in combination with CAR T cells to treat patients who were resistant to or relapsed from previous CAR T cell therapy. Twelve patients with B cell lymphoma with disease progression (n=8) or relapse (n=4) after CD19 CAR T therapy continued to receive pembrolizumab from 3.3 (0.4-42.8) months after CAR T cell infusion, with a 25% optimal ORR (1 CR and 2 PR) (149). The only ≥ grade 3 adverse event was 25% neutropenia. After the first pembrolizumab infusion, CAR T cell re-expansion was detected in 83% of patients. Deep immune profiling demonstrated that, compared with nonresponders, lower-level expression of exhaustion and terminal differentiation markers was found in clinical responders, suggesting that PD-1 blockade is more effective in reversing exhaustion for patients with PD-1 expression under a certain threshold (149). Moreover, the Alexander trial administered CD19/CD22 dual-targeted CAR T cells plus pembrolizumab to 8 diffuse large B cell lymphoma (DLBCL) patients, with 75% ORR and 63% CR. No cases of severe CRS or ICANS were observed (150).

Nivolumab, another anti-PD-1 mAb, was infused into 11 patients with R/R B-NHL three days after CD19 CAR T cell infusion. The ORR and CR rate were 81.81% and 45.45%, respectively (152). Mild CRS was observed in 9 patients, and it could be relieved by glucocorticoids. Although the efficacy of the combination regimen did not appear to be significantly better than that of CD19 CAR T cells alone, it may be associated with a worse baseline of the combination cohort (152). Low-dose nivolumab (1.5 mg/kg) and CD19 CAR T cells were also feasible for follicular lymphoma patients with high PD-1 expression (175).

In ZUMA-6, 4 doses of atezolizumab, an anti-PD-L1 mAb, combined with axicabtagene ciloleucel were administered to 12 patients with R/R DLBCL. CAR T cells were observed to expand robustly, and the ORR of 10 evaluable patients was 90%, including 6 (60%) CR (153). Similarly, another anti-PD-L1 mAb, durvalumab, was infused in 11 patients on Day 29 after CD19 CAR T therapy at a total dose of 1500 mg/4 weeks for 12 months. The optimal ORR was 91% (10/11), and 64% of patients (7/11) achieved CR. Grade 3 or more severe neutropenia and cytopenia occurred in 2 patients, and no CRS was reported (155).

The combination of PD-1/PD-L1 blockade has also been applied in other haematological malignancies. For children with R/R B-ALL, PD-1 inhibition is relatively efficient for those with less persistent CAR T cells and bulk extramedullary disease; however, it is less useful for those with a poor initial response to CAR T cells (151). In terms of MM, BCMA/CS1 bispecific CAR T cells in combination with anti-PD-1 mAb accelerated the clearance of MM cells in mouse models (176). However, prolonged injections of anti-PD-1 mAb after tumour clearance in this study appeared to cause CAR T cell dysfunction, with mice relapsing or failing to prevent tumour outgrowth after tumour rechallenge (176). Further studies are required to determine the optimal duration of PD-1/PD-L1 inhibitor administration to avoid impairing the sustained antitumor potency of CAR T cells.

Together, PD-1/PD-L1 inhibitors appear to be tolerable and can achieve therapeutic improvement of CAR T cell therapy for some patients. However, the overall efficacy is less promising, and for ineffective cases other independent resistance mechanisms deserve further clarification. Other checkpoint inhibitors, such as ipilimumab, a cytotoxic T lymphocyte-associated antigen-4 (CTLA-4) blockade, in combination with CAR T cells are under investigation (NCT00586391). Recently, the utilization of single-cell sequencing facilitated the identification of T cell immunoreceptor with immunoglobulin and immunoreceptor tyrosine-based inhibitory motif domain (TIGIT) as the most notable exhaustion marker of CAR T cells at the transcriptional and protein levels in B-NHL patients. Sequentially, TIGIT blockade was proven to improve the antitumor function of CAR T cells in preclinical studies (156).

### 4-1BB agonists

T cell costimulatory receptors play crucial roles in the CAR molecule construct, and they have also been explored as targets for cancer immunotherapy. CAR T cells with a CD28 costimulatory domain are believed to lead to robust but transient activation, while the signalling of 4-1BB CAR is mild and persistent (177). The addition of exogenous 4-1BB agonists to CAR T cells with the CD28 costimulatory domain is likely to enhance CAR T cell activation, persistence and memory. 4-1BB agonists can also activate NK cells, DCs and other myeloid lineage cells to further boost the immune response (178). Combining HER2-CD28 CAR T cells with an exogenous agonistic 4-1BB monoclonal antibody (mAb) achieved stronger antitumor efficacy and long-term tumour-free survival in a breast tumour mouse model (179). Utomilumab, a 4-1BB agonist entering clinical trials, is being scheduled to be applied for refractory LBCL in the ZUMA-11 trial 1 day following infusion of axicabtagene ciloleucel, an FDA-approved CAR T cell product with the CD28 costimulatory domain, and every 4 weeks for 6 months or until progressive disease (NCT03704298) (180). Data are still being collected.

### Other antibody-based therapies

Antibodies targeting tumour surface antigens are the most prevalent immunotherapy in the treatment of haematological malignancies and have been widely used to bridge CAR T cell therapy. In B-NHL, the CD20 mAbs rituximab and obinutuzumab are the most popular bridging immunotherapies (47). In addition, researchers are exploring their administration concomitantly with CD19 CAR T cells (NCT04002401, NCT04889716). Daratumumab, a first-in-class anti-CD38-mAb for the treatment of MM, is an attractive candidate for cooperating with CAR T cells as it can eliminate MM cells and simultaneously target CD38-positive immunosuppressive cells such as Tregs, regulatory B cells (Bregs) and MDSCs, reversing the suppressive immune microenvironment for CAR T cells (157, 181). Antibody-drug conjugates (ADCs), including the anti-CD30 ADC brentuximab vedotin and the anti-CD79 ADC polatuzumab vedotin for B-NHL and the anti-CD22 antibody inotuzumab ozogamicin, are also used as bridging therapies (10, 158, 159). Bispecific antibodies used in the bridging period include CD20/CD3-targeting mosunetuzumab and glofitamab for B-NHL and CD19/CD3-targeting blinatumomab for B-ALL (160, 164). They can bind to tumour antigens and recruit T cells for accurate killing. Notably, repeated targeting by CAR T cells and antibodies should be highlighted due to the high risk of antigen escape (162, 164). Therefore, close monitoring of antigen expression is needed to design combinations of these immunotherapy agents.

### Combination with different CAR T cell products

Targeting multiple antigens is a pivotal strategy for overcoming antigen-negative relapse. Dual or bispecific CAR T cells are being investigated, while a multitargeted single-CAR design in those products is challenging (182). On the other hand, as clinical applications of multiple single-target CAR T cell products are gradually maturing, combined infusion of different CAR T cell products may achieve clinical translation more easily **(** **Table 4** **)**.

**Table 4:** Clinical trials of CAR T cell cocktail or sequential therapy.

Disease	Authors	Intervention	Targets	N	ORR	CR	Survival	Toxicity
**B-NHL**	Wei et al. (62)	Cocktail	CD19+CD22	66	TP53: 88% Non: 87%	TP53: 50% Non: 45%	NR	CRS: 91%; ICANS: 9%
	Wang et al. (48)	Cocktail	CD19+CD22	38	72%	50%	OS, 55% (1y); PFS, 50% (1y)	CRS: 96%; ICANS: 14%
	Cao et al. (64)	Cocktail	CD19+CD22	42	91%	81%	OS, 83% (2y); PFS, 83% (2y)	CRS: 96%; ICANS: 21%
	Zeng et al. (183)	Cocktail	CD22+CD19	14	71%	50%	OS, 71% (6m); PFS, 50% (6m)	CRS: 93%; GI AE: 29%^a^
**BL**	Liu et al. (184)	Sequential	CD19-CD22-CD20	23	95%	86%	PFS, 78% (18m)	CRS: 70%; ICANS: 22%
**DHL**	Wei et al. (185)	Cocktail	CD19+CD22	12	83%	25%	OS, 75% (1y); PFS, 64% (1y)	CRS: 75%; ICANS: 33%
**B-ALL**	Wang et al. (48)	Cocktail	CD19+CD22	51	96%	96%^c^	OS, 63% (1y); PFS, 53% (1y)	CRS: 96%; ICANS: 14%
	Yan et al. (186)	Cocktail	CD19+CD22	22	100%	100%	OS, 67% (1y); PFS, 59% (1y)	CRS: 87%; GVHD: 30%^b^
	Liu et al. (187)	Sequential	CD19-CD22	21	95%	95%	OS, 89% (1y); EFS, 68% (18m)	CRS: 52%	
	Pan et al. (188)	Sequential	CD19-CD22	20	100%	100%	OS, 92% (1y); LFS, 80% (1y)	CRS: 90%; ICANS: 20%
**MM**	Yan et al. (189)	Cocktail	CD19+BCMA	21	95%	57%	NR	CRS: 90%; ICANS: 10%
	Wang et al. (190)	Cocktail	CD19+BCMA	62	92%	60%	Median PFS: 18.3m	CRS: 95%; ICANS: 11%
	Shi et al. (71)	Cocktail	CD19+BCMA	9	100%	100%	NR	CRS: 100%
	Shi et al. (148)	Cocktail	CD19+BCMA	10	100%	100%	NR	CRS: 100%
	Yan et al. (191)	Cocktail	CD19+BCMA	10	90%	57%^d^	NR	CRS: 100%

NHL, non-Hodgkin lymphoma; BL, Burkitt lymphoma; DHL, double-hit lymphoma; ALL, acute lymphoblastic leukemia; MM, multiple myeloma; ORR, overall response rate; CR, complete response; OS, overall survival; PFS, progression-free survival; EFS, event-free survival; LFS, leukemia-free survival; CRS, cytokine release syndrome; ICANS, immune effector cell-associated neurotoxicity syndrome; NR, not reported; GI AE, gastrointestinal adverse events.

a Patients enrolled in the trial had lymphoma involving the gastrointestinal tract.

b Patients with allo-HSCT history in this trial received allogenic CAR T cells.

c MRD-negative CR/CRi.

d stringent CR.

**Figure 4:**
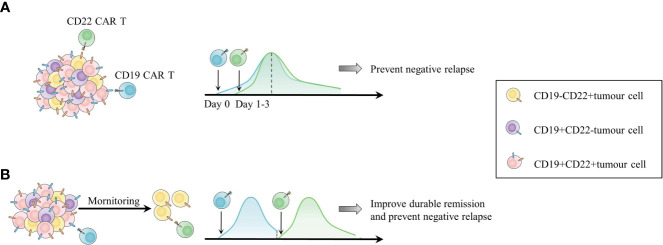
Two combinations of CD19 and CD22 CAR T cell products as an example of combinations among different types of CAR T cells. **(A)** Cocktail infusion of CD19 and CD22 CAR T cells for addressing antigen-negative relapse. **(B)** Sequential infusion of CD19 and CD22 CAR T cells according to the *in vivo* activity of CAR T cells and patient status to overcome both short duration and antigen escape.

The most common combination scheme is cocktail infusion of CD19/CD22 CAR T cells in B cell malignancies **(** **Figure 4** **)** (62, 192). Wang et al. administered CD19 CAR T and CD22 CAR T cells at Day 0 and Day 2, respectively, to B-ALL and B-NHL patients. A total of 94.1% (48/51) of B-ALL patients achieved MRD-negative CR or complete remission with incomplete count recovery (CRi), and 1 patient achieved PR. The median PFS was 13.6 months (95% CI, 6.5-not reached), and the median OS was 31 months (95% CI, 10.6-NR). Among the B-NHL cohorts, 68% (26/38) achieved ORR, and 47% reached CR (192). The median PFS was 9.9 months (95% CI, 3.3-NR), and the median OS was 18.0 months (95% CI, 6.1-NR). The cocktail appears to resolve antigen-negative relapse effectively but still faces the issue of inadequate CAR T cell persistence. Only one patient relapsed with CD19-negative CD22-dim, while 23 patients exhibited CD19+CD22+ relapse. CD19/CD22 CAR T cell cocktail therapy is also promising in improving the outcome of B cell malignancies with the TP53 mutation, which confers a higher rate of CD19-negative relapse and poor prognosis (56, 193). The optimal ORR of 32 TP53-mutated lymphoma patients who received cocktail therapy was similar to that of 34 lymphoma patients without the mutation (87.1% vs. 88.2%, P=0.927) (62). In terms of R/R MM, CD19/BCMA CAR T cell cocktail therapy was designed to extend the target range, covering a proportion of CD19-positive myeloma-like stem cells with drug resistance and propagating characteristics (194). Yan et al. enrolled 21 MM patients and administered CD19 CAR T cells and BCMA CAR T cells simultaneously. Twenty (95%) patients had an overall response, including 9 with stringent CR, 3 with CR, and 5 with VGPR (189).

Sequential infusion is another form of combination. Patients in CR with undetectable CAR T cells are expected to undergo infusion with another type of CAR T cell as maintenance to prolong CAR T cell persistence and prevent antigen-negative relapse. For patients in PR or SD, the second infusion is considered a consolidative regimen to induce further CR (184, 188). Pan et al. infused CD22 CAR T cells into 20 R/R B-ALL patients in CR when the first infusion of CD19 CAR T cells was undetectable, with a median interval of two CAR T cell infusions of 1.65 months. The 12-month LFS was 79.5%, and the 18-month OS rate was 92.3% (188). Moreover, 23 R/R Burkitt lymphoma patients were administered sequential infusions of murine CD19 (mCD19)-humanized CD22 (hCD22)-hCD20 CAR T cells depending on their disease states and *in vivo* CAR T cell lifespan (184). The CR rate was 95% at 3 months after the last infusion. The estimated 18-month CR rate and PFS rate were 78% (95% CI, 54%-91%) and 78% (95% CI, 55%-90%), respectively (184). The toxicities of sequential infusions in these trials were moderate and had no influence on the subsequent CAR T cell infusion. The time interval between infusions should be optimized, as reinfusion too early may impair effective CAR T cells *in vivo* by lymphodepletion, while tumours may recur if there is no timely CAR T cell maintenance. Interestingly, Meng et al. shortened the time interval and did not apply lymphodepletion before the second CAR T cell infusion. They observed re-expansion of the first CAR T cells with unclear mechanisms (195). However, the excessive expansion and activation of CAR T cells raise the concern of aggravated CRS, requiring conscious monitoring in more studies. Other combined CAR T cell products targeting different antigens, such as CD19/CD79b CAR T cells for B-NHL, CD19/CD38, CD19/CD123 CAR T cells for B-ALL, and BCMA/GPRC5D CAR T cells for MM, are being explored (182).

## Discussion

Resistance and relapse are the most prominent challenges of CAR T cell therapy in haematological malignancies, for which searching for combination treatment is a promising strategy for overcoming CAR T cell-centric, tumour-centric and TME-associated hurdles. Chemotherapy and radiotherapy as preconditioning treatments play an important role in subsequent clinical outcomes. In particular, radiotherapy, with its stimulatory function on the immune response, may achieve desired priming of CAR T cell therapy. Head-to-head clinical trials are needed to compare the bridging of radiotherapy with chemotherapy and other emerging bridging therapies. Consolidative allo-HSCT after CAR T cell therapy is a research hotspot among diverse combination modalities of CAR T cell therapy and HSCT. Clarifying indications for consolidative allo-HSCT is crucial to avoiding wasting residual functioning CAR T cells *in vivo*. Targeted therapy and immunotherapy also provide multiple choices to enhance the efficacy of CAR T cell therapy, among which some agents have already been the standard of care for specific haematological tumours, providing safe and reliable therapeutic effects. Patient selection is quite important. Based on genetic testing, targeted therapy is expected to simultaneously eliminate tumours and enhance CAR T cell function. For instance, BCR-ABL-positive patients are likely to benefit more from CAR T cell infusion concomitant with TKI regardless of the turn-off period of CAR T cells. In terms of immunotherapy, there are large interindividual variations in treatment effects, suggesting urgent needs to identify patients who are sensitive to the treatments along with clarifying interactions of the combined agents in a complicated immune microenvironment.

To date, there have been limited clinical data on combinatorial strategies, and more large-scale trials are needed to directly compare them with monotherapy. Simultaneously, more mechanistic studies are expected to further unveil interactions between adjuvant agents and CAR T cells in different differentiation phases, facilitating rational combinatorial regimen design with optimal timing and dosing. As high-throughput screening technologies such as CRISPR screening and single-cell sequencing are utilized to identify more druggable mechanisms and effects of combined agents on CAR T cells, more safe and efficient combination modalities will be discovered to help elicit more potential of the legendary CAR T cell therapy.

## Author contributions

XXiao, ST and YL designed the study. XXiao, YW, ZZ, XW and XXin drafted the manuscript. YW, YY, ZZ and XXiao prepared the tables and figures. All authors participated in the revision of the manuscript. All authors read and approved the final manuscript.

## Funding

This research was supported by grants from the National Natural Science Foundation of China (U2001224); Guangzhou Regenerative Medicine and Health Guangdong Laboratory (2018GZR110105014); the National Key Research and Development Programme of China (2017YFA0105503); Guangdong Students’ Platform for Innovation and Entrepreneurship Training Program (No. S202112121059) and Special Funds for the Cultivation of Guangdong College Students’ Scientific and Technological Innovation (No. pdjh2022b0096).

## Acknowledgments

XXiao, YW, YY and XW are members of the Small Small bird (SSb) group and acknowledge the strong support from SSb. We would like to acknowledge Xinjie Xu, the founder and leader of SSb, for the constructive suggestions of the manuscript. We also sincerely appreciate continuous support from our best friends Sifei Chen and Shengkang Huang.

## Conflict of interest

The authors declare that the research was conducted in the absence of any commercial or financial relationships that could be construed as a potential conflict of interest.

## Publisher’s note

All claims expressed in this article are solely those of the authors and do not necessarily represent those of their affiliated organizations, or those of the publisher, the editors and the reviewers. Any product that may be evaluated in this article, or claim that may be made by its manufacturer, is not guaranteed or endorsed by the publisher.

## Glossary

**Table:**

CAR	chimeric antigen receptor
HSCT	haematopoietic stem cell transplantation
FDA	Food and Drug Administration
R/R	relapsed/refractory
B-ALL	B cell acute lymphoblastic leukaemia
B-NHL	B cell non-Hodgkin lymphoma
MM	multipl e myeloma
TME	tumour microenvironment
scFv	single-chain fragment variable
AICD	activationinduced cell death
MDSCs	myeloid-derived suppressor cells
TAMs	tumourassociated macrophages
Tregs	regulatory T cells
IDO	indoleamine 2,3-dioxygenase
BCMA	B cell maturation antigen
GS	γ-secretase
TRAIL	tumour necrosis factor-related apoptosis-inducing ligand
Bcl-2	B cell lymphoma-2
IAPs	inhibitor of apoptosis family of proteins
OS	overall survival
PD	progressive disease
HR	hazard ratio
CI	confidence interval
Cy	cyclophosphamide
Flu	fludarabine
BSA	body surface area
AUC	area under the curve
LFS	leukaemia-free survival
HL	Hodgkin lymphoma
BTK	Bruton’s tyrosine kinase
allo-HSCT	allogeneic haematological stem cell transplantation
CLL	chronic lymphocytic leukaemia
CR	complete remission
MRD	minimal residual disease
CRS	cytokine release syndrome
GVHD	graft-versus-host disease
DLBCL	diffuse large B cell lymphoma
ORR	overall response rate
ASCT	autologous haematopoietic stem cell transplantation
HDT-ASCT	high-dose chemotherapy and ASCT
BEAM	carmustine etoposide cytarabine and melphalan
PFS	progression-free survival
G-CSF	granulocyte colony-stimulating factor
BUCY	busulfan and cyclophosphamide
VGPR	very good partial remission
PR	partial remission
MCL	mantle cell lymphoma
ITK	IL-2-induced tyrosine kinase
ICANS	immune effector cell-associated neurotoxicity syndrome
PBMCs	peripheral blood mononuclear cells
Th	T helper
mAb	monoclonal antibody
LBCL	large B cell lymphoma
ADC	antibody–drug conjugate
Bregs	regulatory B cells
PD-1	programmed-cell-death-1
PD-L1	programmed-cell-deathligand-1
PI3K	phosphatidylinositol-3-kinase
Tcm	central memory T cell
Tn	naïve T cell
GSI	g-secretase inhibitor
ATRA	all-trans retinoic acid
IL	interleukin
Tscm	T memory stem cell
TGF-β	transforming growth factor-β
COX-2	cyclooxygenase-2
GM-CSF	granulocyte-macrophage colonystimulating factor
DAC	decitabine
DNMTi	DNA methyltransferase inhibitor
AZA	azacitidine
HDACi	histone deacetylase inhibitor
BET	bromodomain and extraterminal domain
cBAF	canonical BRG1/BRMassociated factor
BD98	BRD-K9864598
GSK-3β	glycogen synthase kinase-3β
CTLA-4	cytotoxic T lymphocyte-associated antigen-4
CRi	complete remission with incomplete count recovery
mCD19	murine CD19
hCD22	humanized CD22
TIGIT	T cell immunoreceptor with immunoglobulin and immunoreceptor tyrosine-based inhibitory motif domain
